# Crystal structure of *N*-isopropyl-*N*-(phen­yl)phenyl­glyoxyl­amide

**DOI:** 10.1107/S2056989018013762

**Published:** 2018-10-12

**Authors:** Hisakazu Miyamoto, Genta Yamauchi, Takuya Ueno, Hidehiro Uekusa

**Affiliations:** a Department of Liberal Arts (Sciences & Mathematics), National Institute of, Technology, Kurume College, Fukuoka 830-8555, Japan; b Material Engineering Advanced Course, National Institute of Technology, Kurume College, Fukuoka 830-8555, Japan; c Department of Chemistry, School of Science, Tokyo Institute of Technology, Ookayama 2-12-1-H62, Meguro-ku, Tokyo 152-8551, Japan

**Keywords:** crystal structure, photoreaction

## Abstract

The title compound [systematic name: 2-oxo-*N*,2-diphenyl-*N*-(propan-2-yl)acetamide], C_17_H_17_NO_2_, was synthesized and its photoreactive properties in the crystalline state were investigated. In the mol­ecule, the carbonyl group attached to the phenyl ring adopts an *s*-*trans* configuration with respect to the isopropyl group. Moreover, the distance between the C atom of the carbonyl group and the N-bound C atom of the isopropyl group is 3.845 (2) Å, which is much longer than 3.2 Å, the threshold for photoreactions to take place in the mol­ecule. As a result, the crystal did not photoreact upon UV light irradiation. In the crystal, the mol­ecules are linked *via* weak inter­molecular C—H⋯O hydrogen bonds, forming a layer structure parallel to the *ab* plane.

## Abstract 2

The title compound, C_17_H_17_NO_2_, was synthesized and its photoreactive properties in the crystalline state were investigated. A solid-state photoreaction did not occur because the reaction sites were too far apart in the mol­ecule.

## Chemical context   

**Figure 1:**
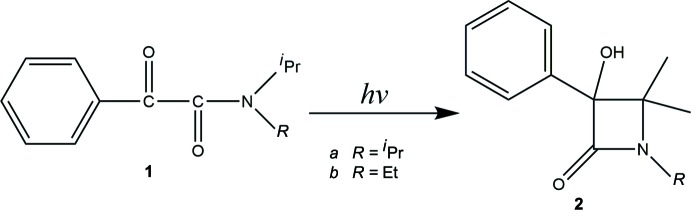
Photoreaction of *N*-isopropyl-phenyl­glyoxyl­amide derivatives.

An achiral mol­ecule of *N*,*N*-diiso­propyl­aryl­glyoxyl­amide **1a** having two isopropyl groups crystallizes in the chiral space group *P*2_1_2_1_2_1_ and is transformed to the optically active β-lactam derivative **2a** upon UV light irradiation (Fig. 1 ▸; Toda *et al.*, 1987 ▸, 1993 ▸; Sekine *et al.*, 1989 ▸; Hashizume *et al.*, 1995 ▸, 1996 ▸, 1998 ▸). Likewise, *N*-eth­yl*-N*-iso­propyl­phenyl­glyoxyl­amide **1b**, having an ethyl group and an isopropyl group, forms a chiral crystal (*P*2_1_2_1_2_1_), and its photoirradiation in the solid state yields the optically active β-lactam derivative **2b** (Fig. 1 ▸; Toda *et al.*, 1997 ▸). Therefore, we synthesized the title compound **1c** having a phenyl group and an isopropyl group, and investigated whether an optically active β-lactam derivative could be obtained by photoreaction of its crystals. It was found that the photoreaction did not proceed in the solid state. In this paper, an explanation for the lack of photoreactivity is presented based on single crystal X-ray structural analysis.

## Structural commentary   

**Figure 2:**
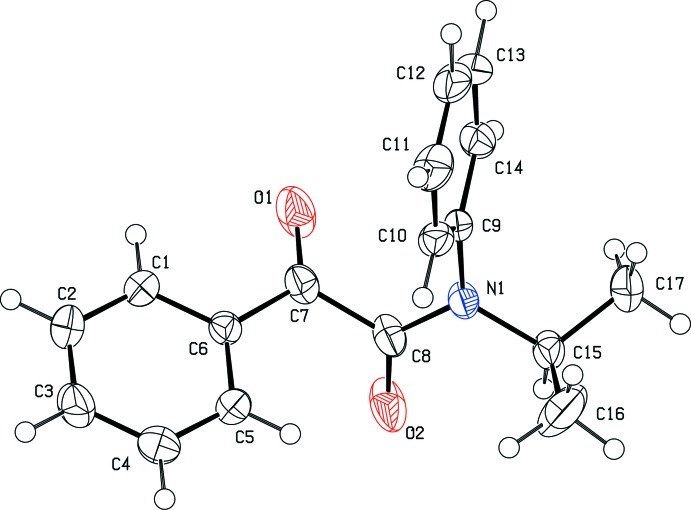
The mol­ecular structure of the title compound **1c**. Displacement ellipsoids for non-H atoms are drawn at the 50% probability level.

In the mol­ecule of **1c**, the carbonyl group (C7=O1) adopts an *s*-*trans* configuration with respect to the isopropyl group (Fig. 2 ▸), in contrast to **1a** and **1b**, which have *s*-*cis* configurations. The torsion angles C7—C8—N1—C15 and O1—C7—C8—O2 are −179.43 (13) and −112.09 (19)°, respectively, in **1c**. The corresponding torsion angles are −5.1 (4) and 88.0 (4)°, respectively, in **1a**, and −10.4 (3) and 90.7 (2)°, respectively, in **1b**; in the case of **1a**, which has two isopropyl groups, the torsion angle including the reacting carbon atom was calculated.
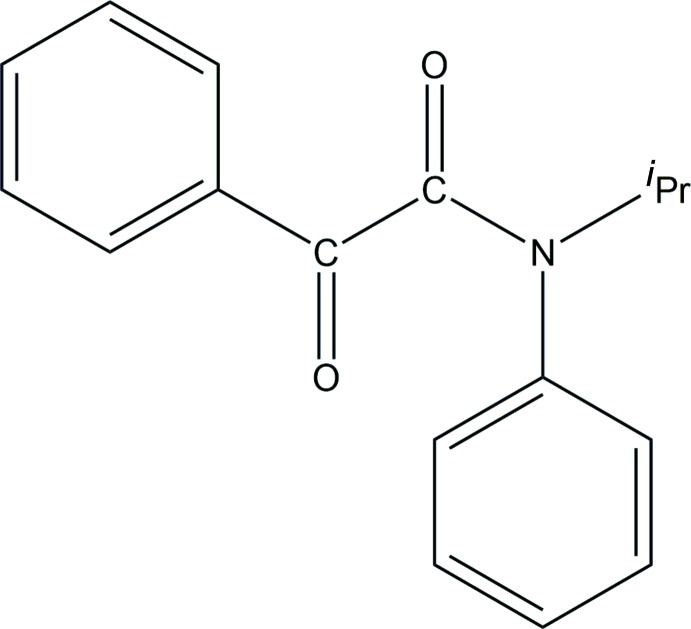


In order for the Norrish–Yang reaction to take place, the reacting atoms in the mol­ecular structure must be in close proximity. In the crystal structure of **1c**, the distance between the γ-hydrogen atom H15 and the carbonyl oxygen atom O1 is 4.565 Å. This inter­atomic distance is much longer than the ideal value of up to about 2.7 Å, at which photoreaction can proceed in the crystal (Konieczny *et al.*, 2018 ▸). Moreover, the distance between the reacting C7 and C15 carbon atoms is 3.845 (2) Å, which is outside the range of ideal values of up to about 3.2 Å. These inter­atomic distances in **1c** are large enough to prevent the photoreaction from taking place. In contrast, the corresponding distances are 2.78 (4) and 2.871 (4) Å in **1a**, and 2.81 (3) and 2.897 (3) Å in **1b**. As those distances are close to the ideal values, the photoreaction could occur in the crystalline state.

## Supra­molecular features   

**Figure 3:**
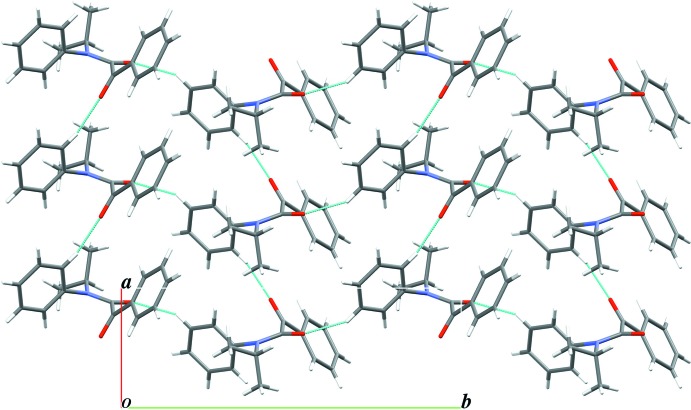
A packing diagram viewed along the *c* axis isfor the title compound **1c**, showing C—H⋯O inter­actions as dotted blue lines.

In the crystal of **1c**, the mol­ecules are linked by weak inter­molecular C—H⋯O inter­actions (C10—H10⋯O1^i^ and C13—H13⋯O2^ii^; symmetry codes as in Table 1 ▸), forming a layer structure parallel to the *ab* plane (Fig. 3 ▸).

## Database survey   

A search of the Cambridge Structural Database (Version 5.39, last update August 2018; Groom *et al.*, 2016 ▸) generates nine hits for compounds based on the *N*-iso­propyl­phenyl­glyoxyl­amide fragment shown in Fig. 1 ▸. These results include five structural analogues including an isopropyl group (JAGLAE; Sekine *et al.*, 1989 ▸), a methacryloyl group (NUKSOB; Sakamoto *et al.*, 1997 ▸), an ethyl group (POWMIX; Toda *et al.*, 1997 ▸), a tigloyl group (WEPCID01; Sakamoto *et al.*, 1997 ▸) and a 2-*tert*-butyl­phenyl group (QUPWEE; Jesuraj & Sivaguru, 2010 ▸). The last compound has a similar mol­ecular structure to that of **1c**, with a corresponding torsion angle of 174.6 (1)°. Of the remaining compounds, three are co-crystals of *N,N*-diiso­prop­yl­aryl­glyoxyl­amide with other organic compounds (ZEDJOH and ZEDJUN; Hashizume *et al.*, 1994 ▸; POWMET; Toda *et al.*, 1997 ▸).

## Synthesis and crystallization   

The title compound was prepared according to a reported method (Toda *et al.*, 1987 ▸,1997 ▸; Sekine *et al.*, 1989 ▸): chlorin­ation of the phenyl­glyoxylic acid with thionyl chloride followed by reaction with *N*-iso­propyl­aniline and tri­ethyl­amine. Thus, to an ice-cooled solution of *N*-iso­propyl­aniline (0.72 ml, 5 mmol) and tri­ethyl­amine (0.70 ml, 5 mmol) in dry diethyl ether (2 ml) was added a solution of benzoyl­formyl chloride (0.84 g, 5 mmol) in dry diethyl ether (2 ml), and the reaction mixture was stirred for 3 h in an ice bath. After filtration of tri­ethyl­ammonium chloride, the filtrate was washed with dilute HCl and aqueous NaHCO_3_ and dried over MgSO_4_. The crude product was recrystallized from benzene to give **1c** as colourless prisms (0.5968 g, 22.4% yield, m.p. 397–401 K); IR (KBr): ν_max_ 1643 and 1681 cm^−1^; ^1^H NMR (CDCl_3_): δ_H_ 1.21 (*d*, 6H, CH*Me*_2_), 5.10 (*sep*, 1H, N—C*H*), 7.07–7.80 (*m*, 10H, Ar*H*). Single crystals of **1c** suitable for X-ray diffraction were grown from a benzene solution.

## Photoreaction in the solid state   

**1c** (51.3 mg, 0.21 mmol) was pulverized in a mortar and irradiated with a 400 W high pressure mercury lamp for 20 h. No reaction took place, as determined by TLC, IR and NMR spectroscopy.

## Refinement   

Crystal data, data collection and structure refinement details are summarized in Table 2 ▸. All H atoms were positioned in geometrically calculated positions (C—H = 0.95–0.98 Å) and refined using a riding model with *U*_iso_(H) = 1.2*U*_eq_(C) and 1.5*U*_eq_(C-meth­yl).

## Supplementary Material

Crystal structure: contains datablock(s) I. DOI: 10.1107/S2056989018013762/is5500sup1.cif

Structure factors: contains datablock(s) I. DOI: 10.1107/S2056989018013762/is5500Isup2.hkl

Supporting information file. DOI: 10.1107/S2056989018013762/is5500Isup3.cml

CCDC reference: 1870320

Additional supporting information:  crystallographic information; 3D view; checkCIF report

## Figures and Tables

**Table 1:** Hydrogen-bond geometry (Å, °)

*D*—H⋯*A*	*D*—H	H⋯*A*	*D*⋯*A*	*D*—H⋯*A*
C10—H10⋯O1^i^	0.95	2.32	3.2140 (18)	157
C13—H13⋯O2^ii^	0.95	2.48	3.2895 (18)	143

Symmetry codes: (i) 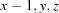; (ii) 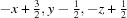.

**Table 2:** Experimental details

Crystal data	
Chemical formula	C_17_H_17_NO_2_
*M*_r_	267.31
Crystal system, space group	Monoclinic, *P*2_1_/*n*
Temperature (K)	93
*a*, *b*, *c* (Å)	5.8354 (5), 16.5123 (14), 15.1330 (12)
β (°)	93.837 (2)
*V* (Å^3^)	1454.9 (2)
*Z*	4
Radiation type	Mo *K*α
μ (mm^−1^)	0.08
Crystal size (mm)	0.25 × 0.18 × 0.14

Data collection	
Diffractometer	Rigaku R-AXIS RAPID
Absorption correction	Multi-scan (*ABSCOR*; Higashi, 1995 ▸)
*T*_min_, *T*_max_	0.642, 0.989
No. of measured, independent and observed [*I* > 2σ(*I*)] reflections	13857, 3316, 2572
*R*_int_	0.043
(sin θ/λ)_max_ (Å^−1^)	0.648

Refinement	
*R*[*F* ^2^ > 2σ(*F* ^2^)], *wR*(*F* ^2^), *S*	0.054, 0.143, 1.13
No. of reflections	3316
No. of parameters	183
H-atom treatment	H-atom parameters constrained
Δρ_max_, Δρ_min_ (e Å^−3^)	0.36, −0.21

Computer programs: *RAPID-AUTO* (Rigaku, 1998 ▸), *SHELXT2014* (Sheldrick, 2015*a* ▸), *SHELXL2018* (Sheldrick, 2015*b* ▸), *Mercury* (Macrae *et al.*, 2006 ▸) and *publCIF* (Westrip, 2010 ▸).

## supplementary crystallographic information

### Crystal data

**Table:**

C_17_H_17_NO_2_	*F*(000) = 568
*M**_r_* = 267.31	*D*_x_ = 1.220 Mg m^−^^3^
Monoclinic, *P*2_1_/*n*	Mo *K*α radiation, λ = 0.71075 Å
*a* = 5.8354 (5) Å	Cell parameters from 13857 reflections
*b* = 16.5123 (14) Å	θ = 3.7–27.5°
*c* = 15.1330 (12) Å	µ = 0.08 mm^−^^1^
β = 93.837 (2)°	*T* = 93 K
*V* = 1454.9 (2) Å^3^	Block, colorless
*Z* = 4	0.25 × 0.18 × 0.14 mm

### Data collection

**Table:**

Rigaku R-AXIS RAPID diffractometer	3316 independent reflections
Radiation source: rotating anode X-ray	2572 reflections with *I* > 2σ(*I*)
Detector resolution: 10.0 pixels mm^-1^	*R*_int_ = 0.043
ω–scan	θ_max_ = 27.5°, θ_min_ = 3.7°
Absorption correction: multi-scan (ABSCOR; Higashi, 1995)	*h* = −7→6
*T*_min_ = 0.642, *T*_max_ = 0.989	*k* = −21→21
13857 measured reflections	*l* = −19→19

### Refinement

**Table:**

Refinement on *F*^2^	0 restraints
Least-squares matrix: full	Hydrogen site location: inferred from neighbouring sites
*R*[*F*^2^ > 2σ(*F*^2^)] = 0.054	H-atom parameters constrained
*wR*(*F*^2^) = 0.143	*w* = 1/[σ^2^(*F*_o_^2^) + (0.0737*P*)^2^ + 0.1802*P*] where *P* = (*F*_o_^2^ + 2*F*_c_^2^)/3
*S* = 1.13	(Δ/σ)_max_ < 0.001
3316 reflections	Δρ_max_ = 0.36 e Å^−^^3^
183 parameters	Δρ_min_ = −0.21 e Å^−^^3^

### Special details

**Table:**

Geometry. All esds (except the esd in the dihedral angle between two l.s. planes) are estimated using the full covariance matrix. The cell esds are taken into account individually in the estimation of esds in distances, angles and torsion angles; correlations between esds in cell parameters are only used when they are defined by crystal symmetry. An approximate (isotropic) treatment of cell esds is used for estimating esds involving l.s. planes.

### Fractional atomic coordinates and isotropic or equivalent isotropic displacement parameters (Å^2^)

**Table:**

	*x*	*y*	*z*	*U*_iso_*/*U*_eq_	
O1	0.9019 (2)	0.43953 (8)	0.30276 (9)	0.0606 (4)	
O2	0.6189 (3)	0.53493 (7)	0.14588 (8)	0.0580 (4)	
N1	0.5441 (2)	0.40017 (7)	0.15732 (7)	0.0296 (3)	
C1	0.7452 (3)	0.54690 (9)	0.42792 (9)	0.0349 (3)	
H1	0.890380	0.521888	0.440442	0.042*	
C2	0.6582 (3)	0.59840 (10)	0.48934 (10)	0.0419 (4)	
H2	0.742860	0.608701	0.544010	0.050*	
C3	0.4469 (3)	0.63490 (10)	0.47073 (11)	0.0444 (4)	
H3	0.386515	0.670465	0.512701	0.053*	
C4	0.3231 (3)	0.61973 (10)	0.39107 (11)	0.0416 (4)	
H4	0.178359	0.645041	0.378623	0.050*	
C5	0.4093 (2)	0.56796 (9)	0.32967 (9)	0.0324 (3)	
H5	0.323566	0.557451	0.275295	0.039*	
C6	0.6215 (2)	0.53134 (8)	0.34761 (9)	0.0270 (3)	
C7	0.7246 (2)	0.47610 (8)	0.28442 (10)	0.0328 (3)	
C8	0.6187 (3)	0.47218 (8)	0.18929 (10)	0.0341 (3)	
C9	0.5209 (2)	0.33160 (7)	0.21501 (8)	0.0244 (3)	
C10	0.3298 (2)	0.32551 (9)	0.26361 (9)	0.0306 (3)	
H10	0.216281	0.366815	0.260071	0.037*	
C11	0.3046 (3)	0.25880 (10)	0.31762 (9)	0.0424 (4)	
H11	0.173330	0.254294	0.351177	0.051*	
C12	0.4701 (3)	0.19876 (9)	0.32281 (10)	0.0463 (4)	
H12	0.451759	0.152802	0.359426	0.056*	
C13	0.6617 (3)	0.20564 (9)	0.27481 (11)	0.0444 (4)	
H13	0.776465	0.164769	0.279285	0.053*	
C14	0.6880 (2)	0.27157 (9)	0.22024 (10)	0.0345 (3)	
H14	0.819139	0.275831	0.186591	0.041*	
C15	0.4431 (3)	0.39643 (8)	0.06438 (9)	0.0357 (4)	
H15	0.509044	0.442544	0.031566	0.043*	
C16	0.1885 (4)	0.40866 (18)	0.06136 (13)	0.0788 (8)	
H16A	0.128132	0.413824	−0.000383	0.118*	
H16B	0.154033	0.458040	0.093861	0.118*	
H16C	0.116587	0.362105	0.088643	0.118*	
C17	0.5082 (4)	0.31954 (11)	0.01865 (11)	0.0496 (5)	
H17A	0.454587	0.322180	−0.044054	0.074*	
H17B	0.436796	0.273184	0.046472	0.074*	
H17C	0.675558	0.313261	0.023782	0.074*	

### Atomic displacement parameters (Å^2^)

**Table:**

	*U*^11^	*U*^22^	*U*^33^	*U*^12^	*U*^13^	*U*^23^
O1	0.0388 (7)	0.0666 (9)	0.0745 (9)	0.0178 (6)	−0.0104 (6)	−0.0379 (7)
O2	0.1117 (11)	0.0272 (6)	0.0356 (6)	−0.0214 (6)	0.0097 (7)	−0.0026 (5)
N1	0.0430 (7)	0.0230 (6)	0.0231 (5)	−0.0042 (5)	0.0039 (5)	−0.0016 (4)
C1	0.0364 (8)	0.0353 (7)	0.0318 (7)	0.0036 (6)	−0.0059 (6)	−0.0020 (6)
C2	0.0518 (9)	0.0446 (9)	0.0286 (7)	0.0014 (7)	−0.0024 (7)	−0.0067 (6)
C3	0.0558 (10)	0.0418 (9)	0.0365 (8)	0.0068 (7)	0.0099 (7)	−0.0095 (7)
C4	0.0378 (8)	0.0411 (8)	0.0458 (9)	0.0109 (6)	0.0025 (7)	−0.0034 (7)
C5	0.0344 (7)	0.0327 (7)	0.0294 (7)	0.0003 (6)	−0.0029 (6)	0.0005 (5)
C6	0.0313 (7)	0.0229 (6)	0.0267 (6)	−0.0019 (5)	0.0017 (6)	0.0001 (5)
C7	0.0318 (7)	0.0289 (7)	0.0379 (8)	−0.0024 (5)	0.0025 (6)	−0.0080 (6)
C8	0.0470 (9)	0.0261 (7)	0.0301 (7)	−0.0068 (6)	0.0103 (6)	−0.0050 (5)
C9	0.0296 (7)	0.0215 (6)	0.0216 (6)	−0.0004 (5)	−0.0011 (5)	−0.0024 (5)
C10	0.0318 (7)	0.0350 (7)	0.0250 (6)	0.0047 (5)	0.0023 (6)	0.0006 (5)
C11	0.0506 (9)	0.0487 (9)	0.0283 (7)	−0.0114 (7)	0.0058 (7)	0.0063 (7)
C12	0.0755 (12)	0.0301 (8)	0.0312 (8)	−0.0093 (7)	−0.0121 (8)	0.0097 (6)
C13	0.0600 (11)	0.0278 (7)	0.0427 (8)	0.0139 (7)	−0.0162 (8)	−0.0033 (6)
C14	0.0325 (7)	0.0347 (7)	0.0359 (7)	0.0066 (6)	−0.0003 (6)	−0.0067 (6)
C15	0.0603 (10)	0.0256 (7)	0.0211 (6)	−0.0041 (6)	0.0022 (6)	0.0004 (5)
C16	0.0715 (14)	0.128 (2)	0.0338 (9)	0.0442 (14)	−0.0149 (9)	−0.0091 (11)
C17	0.0712 (12)	0.0450 (9)	0.0318 (8)	0.0037 (8)	−0.0030 (8)	−0.0123 (7)

### Geometric parameters (Å, º)

**Table:**

O1—C7	1.2141 (18)	C9—C14	1.3890 (18)
O2—C8	1.2269 (18)	C10—C11	1.385 (2)
N1—C8	1.3451 (17)	C10—H10	0.9500
N1—C9	1.4417 (16)	C11—C12	1.382 (2)
N1—C15	1.4893 (17)	C11—H11	0.9500
C1—C2	1.381 (2)	C12—C13	1.378 (3)
C1—C6	1.3952 (19)	C12—H12	0.9500
C1—H1	0.9500	C13—C14	1.381 (2)
C2—C3	1.385 (2)	C13—H13	0.9500
C2—H2	0.9500	C14—H14	0.9500
C3—C4	1.386 (2)	C15—C16	1.497 (3)
C3—H3	0.9500	C15—C17	1.507 (2)
C4—C5	1.382 (2)	C15—H15	1.0000
C4—H4	0.9500	C16—H16A	0.9800
C5—C6	1.3883 (19)	C16—H16B	0.9800
C5—H5	0.9500	C16—H16C	0.9800
C6—C7	1.4781 (19)	C17—H17A	0.9800
C7—C8	1.529 (2)	C17—H17B	0.9800
C9—C10	1.3796 (19)	C17—H17C	0.9800
			
C8—N1—C9	121.17 (11)	C11—C10—H10	120.2
C8—N1—C15	118.33 (11)	C12—C11—C10	120.15 (15)
C9—N1—C15	119.47 (10)	C12—C11—H11	119.9
C2—C1—C6	120.50 (14)	C10—C11—H11	119.9
C2—C1—H1	119.8	C13—C12—C11	119.95 (14)
C6—C1—H1	119.8	C13—C12—H12	120.0
C1—C2—C3	119.57 (14)	C11—C12—H12	120.0
C1—C2—H2	120.2	C12—C13—C14	120.43 (14)
C3—C2—H2	120.2	C12—C13—H13	119.8
C2—C3—C4	120.23 (14)	C14—C13—H13	119.8
C2—C3—H3	119.9	C13—C14—C9	119.41 (14)
C4—C3—H3	119.9	C13—C14—H14	120.3
C5—C4—C3	120.31 (14)	C9—C14—H14	120.3
C5—C4—H4	119.8	N1—C15—C16	110.62 (13)
C3—C4—H4	119.8	N1—C15—C17	111.89 (12)
C4—C5—C6	119.86 (13)	C16—C15—C17	112.32 (16)
C4—C5—H5	120.1	N1—C15—H15	107.2
C6—C5—H5	120.1	C16—C15—H15	107.2
C5—C6—C1	119.53 (13)	C17—C15—H15	107.2
C5—C6—C7	122.58 (12)	C15—C16—H16A	109.5
C1—C6—C7	117.90 (12)	C15—C16—H16B	109.5
O1—C7—C6	122.44 (13)	H16A—C16—H16B	109.5
O1—C7—C8	118.59 (13)	C15—C16—H16C	109.5
C6—C7—C8	118.61 (12)	H16A—C16—H16C	109.5
O2—C8—N1	124.45 (13)	H16B—C16—H16C	109.5
O2—C8—C7	116.97 (12)	C15—C17—H17A	109.5
N1—C8—C7	118.47 (12)	C15—C17—H17B	109.5
C10—C9—C14	120.44 (13)	H17A—C17—H17B	109.5
C10—C9—N1	119.48 (11)	C15—C17—H17C	109.5
C14—C9—N1	120.07 (12)	H17A—C17—H17C	109.5
C9—C10—C11	119.61 (13)	H17B—C17—H17C	109.5
C9—C10—H10	120.2		
			
C6—C1—C2—C3	−0.2 (2)	O1—C7—C8—N1	64.3 (2)
C1—C2—C3—C4	0.2 (3)	C6—C7—C8—N1	−122.42 (15)
C2—C3—C4—C5	0.1 (3)	C8—N1—C9—C10	80.08 (17)
C3—C4—C5—C6	−0.4 (2)	C15—N1—C9—C10	−88.16 (15)
C4—C5—C6—C1	0.4 (2)	C8—N1—C9—C14	−101.03 (16)
C4—C5—C6—C7	−179.31 (14)	C15—N1—C9—C14	90.73 (16)
C2—C1—C6—C5	−0.1 (2)	C14—C9—C10—C11	−0.3 (2)
C2—C1—C6—C7	179.61 (14)	N1—C9—C10—C11	178.59 (12)
C5—C6—C7—O1	−174.46 (15)	C9—C10—C11—C12	0.1 (2)
C1—C6—C7—O1	5.9 (2)	C10—C11—C12—C13	0.6 (2)
C5—C6—C7—C8	12.5 (2)	C11—C12—C13—C14	−1.1 (2)
C1—C6—C7—C8	−167.15 (13)	C12—C13—C14—C9	0.9 (2)
C9—N1—C8—O2	−171.72 (15)	C10—C9—C14—C13	−0.2 (2)
C15—N1—C8—O2	−3.3 (2)	N1—C9—C14—C13	−179.05 (12)
C9—N1—C8—C7	12.2 (2)	C8—N1—C15—C16	−91.50 (19)
C15—N1—C8—C7	−179.43 (13)	C9—N1—C15—C16	77.08 (19)
O1—C7—C8—O2	−112.09 (19)	C8—N1—C15—C17	142.45 (15)
C6—C7—C8—O2	61.20 (19)	C9—N1—C15—C17	−48.97 (18)

### Hydrogen-bond geometry (Å, º)

**Table:**

*D*—H···*A*	*D*—H	H···*A*	*D*···*A*	*D*—H···*A*
C10—H10···O1^i^	0.95	2.32	3.2140 (18)	157
C13—H13···O2^ii^	0.95	2.48	3.2895 (18)	143

Symmetry codes: (i) *x*−1, *y*, *z*; (ii) −*x*+3/2, *y*−1/2, −*z*+1/2.
